# Supplementary material for: Modeling glioblastoma heterogeneity as a dynamic network of cell states
Source: Mol Syst Biol. 2021 Sep 16;17(9):e10105. doi: 10.15252/msb.202010105 (PMC8444284; doi:10.15252/msb.202010105)
Supplement: Supplementary file 6 — Source Data for Figure 5 [file MSB-17-e10105-s004.zip › Figure5A_sourcedata/GSEA_3071/hallmarks_stateIII.GseaPreranked.1623742540963/HALLMARK_MYC_TARGETS_V1.html]

Details for gene set HALLMARK\_MYC\_TARGETS\_V1[GSEA]

|  || Dataset | state33071 |
| Phenotype | NoPhenotypeAvailable |
| Upregulated in class | na\_pos |
| GeneSet | HALLMARK\_MYC\_TARGETS\_V1 |
| Enrichment Score (ES) | 0.7603103 |
| Normalized Enrichment Score (NES) | 7.4114957 |
| Nominal p-value | 0.0 |
| FDR q-value | 0.0 |
| FWER p-Value | 0.0 |
Table: GSEA Results Summary

  

Fig 1: Enrichment plot: HALLMARK\_MYC\_TARGETS\_V1      
 Profile of the Running ES Score & Positions of GeneSet Members on the Rank Ordered List

  

| PROBE | GENE SYMBOL | GENE\_TITLE | RANK IN GENE LIST | RANK METRIC SCORE | RUNNING ES | CORE ENRICHMENT || 1 | RPLP0 |  |  | 6 | 0.633 | 0.0299 | Yes |
| 2 | RPL6 |  |  | 16 | 0.555 | 0.0552 | Yes |
| 3 | RPL22 |  |  | 22 | 0.520 | 0.0798 | Yes |
| 4 | EEF1B2 |  |  | 28 | 0.501 | 0.1034 | Yes |
| 5 | SNRPD2 |  |  | 29 | 0.500 | 0.1283 | Yes |
| 6 | EIF2S2 |  |  | 30 | 0.499 | 0.1532 | Yes |
| 7 | RPS6 |  |  | 41 | 0.480 | 0.1745 | Yes |
| 8 | RPL14 |  |  | 42 | 0.479 | 0.1983 | Yes |
| 9 | RPS5 |  |  | 57 | 0.454 | 0.2173 | Yes |
| 10 | RPS10 |  |  | 60 | 0.452 | 0.2392 | Yes |
| 11 | PSMA7 |  |  | 78 | 0.435 | 0.2565 | Yes |
| 12 | YWHAE |  |  | 85 | 0.427 | 0.2762 | Yes |
| 13 | NPM1 |  |  | 88 | 0.421 | 0.2966 | Yes |
| 14 | RPS3 |  |  | 90 | 0.420 | 0.3173 | Yes |
| 15 | VDAC1 |  |  | 98 | 0.413 | 0.3360 | Yes |
| 16 | RPL34 |  |  | 105 | 0.407 | 0.3547 | Yes |
| 17 | EIF3J |  |  | 110 | 0.403 | 0.3737 | Yes |
| 18 | PPIA |  |  | 121 | 0.395 | 0.3908 | Yes |
| 19 | PABPC1 |  |  | 123 | 0.394 | 0.4102 | Yes |
| 20 | COX5A |  |  | 131 | 0.389 | 0.4277 | Yes |
| 21 | SRM |  |  | 133 | 0.388 | 0.4468 | Yes |
| 22 | EIF4A1 |  |  | 150 | 0.374 | 0.4613 | Yes |
| 23 | SSBP1 |  |  | 156 | 0.370 | 0.4784 | Yes |
| 24 | GLO1 |  |  | 161 | 0.367 | 0.4956 | Yes |
| 25 | SET |  |  | 163 | 0.366 | 0.5135 | Yes |
| 26 | NME1 |  |  | 166 | 0.364 | 0.5311 | Yes |
| 27 | PSMB3 |  |  | 171 | 0.361 | 0.5481 | Yes |
| 28 | RPL18 |  |  | 178 | 0.355 | 0.5642 | Yes |
| 29 | SNRPG |  |  | 186 | 0.349 | 0.5797 | Yes |
| 30 | UBE2L3 |  |  | 216 | 0.331 | 0.5887 | Yes |
| 31 | RANBP1 |  |  | 221 | 0.328 | 0.6040 | Yes |
| 32 | RPS2 |  |  | 228 | 0.325 | 0.6186 | Yes |
| 33 | NHP2 |  |  | 232 | 0.322 | 0.6338 | Yes |
| 34 | ACP1 |  |  | 249 | 0.310 | 0.6452 | Yes |
| 35 | PTGES3 |  |  | 252 | 0.310 | 0.6600 | Yes |
| 36 | IMPDH2 |  |  | 253 | 0.309 | 0.6754 | Yes |
| 37 | HNRNPA1 |  |  | 270 | 0.302 | 0.6864 | Yes |
| 38 | C1QBP |  |  | 277 | 0.297 | 0.6996 | Yes |
| 39 | HSPE1 |  |  | 279 | 0.297 | 0.7141 | Yes |
| 40 | POLE3 |  |  | 301 | 0.290 | 0.7231 | Yes |
| 41 | PA2G4 |  |  | 308 | 0.289 | 0.7360 | Yes |
| 42 | SNRPD1 |  |  | 376 | 0.268 | 0.7320 | Yes |
| 43 | RAN |  |  | 388 | 0.264 | 0.7423 | Yes |
| 44 | EIF4H |  |  | 389 | 0.264 | 0.7555 | Yes |
| 45 | SERBP1 |  |  | 420 | 0.253 | 0.7603 | Yes |
| 46 | CDC45 |  |  | 506 | -0.255 | 0.7511 | No |
| 47 | MYC |  |  | 599 | -0.260 | 0.7402 | No |
| 48 | MCM4 |  |  | 638 | -0.261 | 0.7434 | No |
| 49 | PCNA |  |  | 1116 | -0.286 | 0.6346 | No |
| 50 | CSTF2 |  |  | 2134 | -0.344 | 0.3893 | No |
| 51 | CANX |  |  | 2209 | -0.349 | 0.3876 | No |
| 52 | MCM2 |  |  | 2303 | -0.357 | 0.3814 | No |
| 53 | U2AF1 |  |  | 2460 | -0.370 | 0.3596 | No |
| 54 | CCNA2 |  |  | 2635 | -0.385 | 0.3338 | No |
Table: GSEA details [plain text format]

  

Fig 2: HALLMARK\_MYC\_TARGETS\_V1: Random ES distribution      
 Gene set null distribution of ES for **HALLMARK\_MYC\_TARGETS\_V1**
